# Parametric Study of the Effect of Anchor Drive Bolt Geometry on Stress Distribution and Direction of Crack Formation in the Rock Medium

**DOI:** 10.3390/ma18174136

**Published:** 2025-09-03

**Authors:** Józef Jonak, Robert Karpiński, Andrzej Wójcik

**Affiliations:** Department of Machine Design and Mechatronics, Faculty of Mechanical Engineering, Lublin University of Technology, ul. Nadbystrzycka 36, 20-618 Lublin, Poland

**Keywords:** undercutting anchor for rock detachment, drive bolt termination, anchor hole bottom geometry, finite element method (FEM), crack propagation, rock fragment separation

## Abstract

This paper presents an analysis of the influence of the termination geometry of an undercutting anchor drive bolt and the shape of the bottom of the anchor hole on the initiation and progression of failure processes in a rock medium. The study employed the finite element method (FEM) to model various bolt termination configurations, including cylindrical terminations with a 2 × 2 mm chamfer, a rounded termination with radius *R*, and a conical termination. The interaction of these bolt geometries with both cylindrical and conical hole bottoms was analyzed. The numerical simulations enabled the identification of stress concentration zones and crack propagation paths, which are critical to understanding the efficiency and mechanism of rock failure. The results indicate that the geometry of the bolt termination significantly influences stress distribution within the contact zone, as well as the extent and morphology of the resulting failure zone. Specifically, employing a cylindrical termination with a 2 × 2 mm chamfer in combination with a conical hole bottom promotes the development of deep fractures, which may lead to the detachment of larger rock fragments. This mechanism may be useful in the development of non-explosive rock fragmentation technologies. The findings provide a foundation for further optimization of anchor designs and the development of targeted excavation methods in mining and geotechnical engineering.

## 1. Introduction

Engineering anchors represent a crucial component of fastening systems used across a broad spectrum of technical disciplines—from general construction and civil engineering to mining; geotechnics; and the offshore sector [1,2,3,4,5]. They serve as load-transferring elements between structural components and substrate materials such as concrete, masonry, or rock masses. Depending on design requirements and operating conditions, various types of anchors are employed, including bonded, mechanical, expansion, and undercut anchors [6,7,8]. The latter operate based on mechanical interlock within a specially prepared undercut in the substrate. The diversity in anchor design and the wide range of installation methods enable their effective application under static and dynamic loading conditions, as well as in aggressive environments [9,10,11].

A particularly important category of anchors includes systems designed for use in rock masses, which are characterized by significant structural heterogeneity and anisotropy of mechanical properties [12,13,14]. In such conditions, standard anchoring methods may fail to provide sufficient load-bearing capacity and reliability, prompting the development of advanced anchoring technologies employing components with specialized geometries—such as undercutting head bolts or expansion anchors with additional spreading mechanisms. Anchors are also used beyond the traditional scope of civil engineering [15,16,17,18,19,20], including in systems for stabilizing offshore drilling platforms [21,22,23], support structures for renewable energy installations, and innovative technologies for rock fragmentation and detachment of rock blocks [24,25,26].

In the context of research and development, particular attention is directed toward the load transfer mechanism of undercutting anchors and their influence on the initiation and propagation of the failure zone within the surrounding medium. These issues are critical both for the design of reliable fixings in concrete and rock and for the advancement of novel, non-explosive rock fragmentation methods under challenging mining conditions [27,28,29]. This study aims to analyze the effect of the drive bolt termination geometry of an undercutting anchor and the shape of the anchor hole bottom on stress distribution and crack propagation trajectories in a rock medium. The findings may serve as a basis for the optimization of advanced structural solutions in rock detachment technologies [30,31,32,33].

In recent years, the use of anchorage systems has attracted increasing attention, particularly in the context of retrofitting and structural reinforcement of historical buildings and existing foundations. These systems are widely recognized for their ability to enhance the structural integrity and prolong the lifespan of aging or deteriorated constructions, contributing significantly to their preservation and stability [21,22,23,34,35].

**Figure 1:**
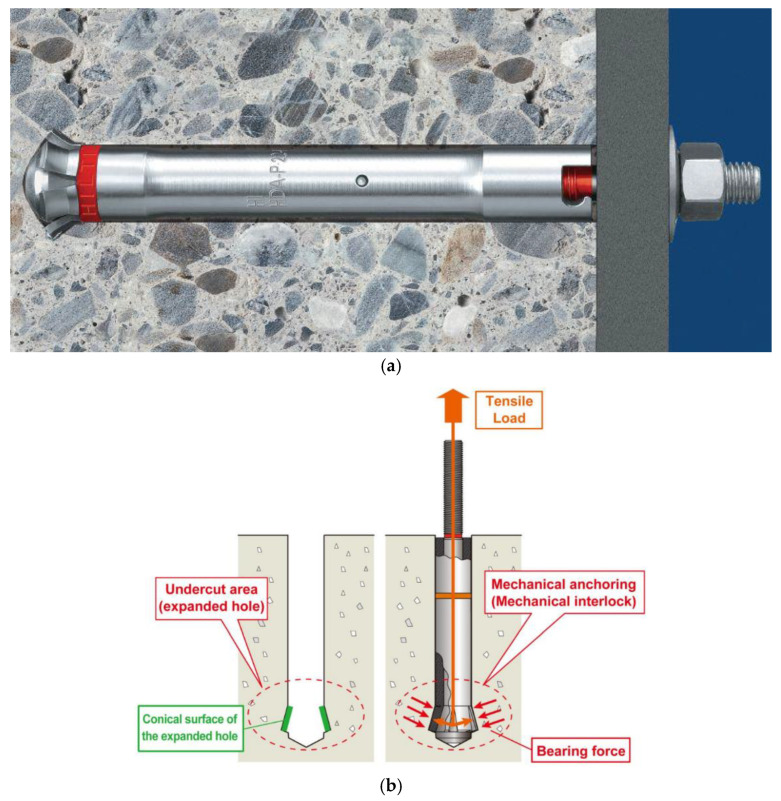
(**a**) HDA-P type undercut anchor; (**b**) Principles of anchoring of an ANZEX bolt.

In typical applications, steel anchors serve as essential structural components for securing infrastructure elements to concrete structures, masonry, or rock masses. Various structural types and installation methods are available, including cast-in-place anchors (e.g., headed bolts) embedded during concrete pouring, mechanically installed post-installed anchors [36], and bonded anchors using appropriate polymer adhesives [37,38]. The installation principle of a typical mechanically installed undercut anchor is illustrated in Figure 1 [39,40].

Anchor fastening systems in concrete typically fail due to concrete-related damage. Depending on the type of loading (tensile or shear), several failure modes can be distinguished, including anchor pull-out, steel rod failure, side-face blowout, concrete cone breakout, splitting failure, or a combination of concrete and anchor failure under shear loads [37,38]. Transitions between these failure modes are governed by several parameters, such as concrete strength and embedment depth [41,42,43].

**Figure 2:**
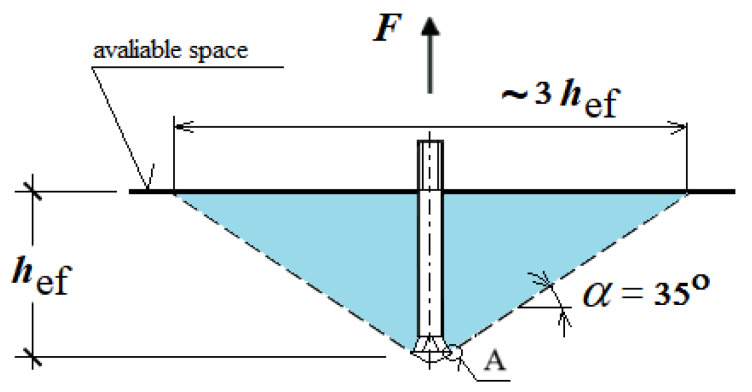
Simplified model of the failure zone in the medium under the action of an undercutting anchor: A—location of the crack initiation point.

Concrete failure in the vicinity of the anchor is commonly characterized by a fracture resembling a conical breakout shape [44,45,46,47] (Figure 2). The crack initiation point (point A) of the surface fissure leading to the formation of the failure cone is typically located at the outer edge of the anchor head (at the base of the conical portion). Such a failure mechanism is observed in the majority of cases involving mechanically installed anchors.

Fracture is considered a brittle failure, characterized by a sudden drop in the load–displacement curve after the peak load, caused by rapid and unstable crack propagation in concrete. Eligehausen et al. [48,49] reported that circumferential cracking during the failure of the concrete cone primarily depends on the effective embedment depth (*h*_ef_) of the anchor. To date, numerous studies [44,45,46,50,51] have been conducted on anchors subjected to tensile loads, as it is essential to investigate the influence of various design and technological parameters on the behavior of the anchor–concrete system, its evaluation, and design procedures [47]. Detailed investigations on the influence of geometric parameters of cast-in-place anchors, including headed bolts, can be found in [52].

Recently, particular research attention has focused on the development of anchor load-bearing capacity under variable (cyclic or dynamic) loading conditions [53], which pose a serious threat to the long-term performance of anchorage systems, especially in regions affected by increased tectonic activity.

In contrast, issues related to anchor installation in rock masses are highly complex [37], primarily due to the significant heterogeneity of the rock structure [54].

In such conditions, chemically bonded anchors may offer a viable solution [12,55]; however, given the scope of this study, they were not subjected to detailed analysis. Other application areas for mechanically installed anchors include, for example, anchoring systems used to stabilize offshore drilling platforms on the seabed [56,57].

The aspect of medium fracturing under tensile forces applied to the anchor [44,48,49,50] is closely related to the topic addressed in this article, as it pertains to the proposed use of undercutting anchors in rock detachment technologies [58,59]. This technology is currently being tested by members of the research team as an alternative to conventional excavation methods, particularly in challenging environments such as mining operations, where mechanical or explosive rock fragmentation is not feasible.

Typical mechanical excavation technologies [60,61,62] are based on the use of excavating machines equipped with cutting tools such as discs or rotary knives [63,64,65]. These technologies enable high-performance coal and rock excavation, as well as the tunneling of large-diameter or large-cross-section openings. Depending on the local excavation conditions, both the geometry of the tools and their arrangement on the cutting heads are optimized. These tasks are carried out using numerical methods such as the finite element method (FEM) [66,67,68] or the discrete element method (DEM) [69,70,71]. Reference [72] presents numerical approaches for modeling rock–tool interactions, including simulations using the discrete element method (DEM), smoothed particle hydrodynamics (SPH), and predictive models enhanced with machine learning (ML). The study evaluates cutting process optimization strategies that employ topology optimization to balance objectives such as energy efficiency, chip formation control, and tool lifespan.

The fracture mechanics of the medium—whether occurring during anchor installation in concrete structures or during the proposed rock block separation—share many fundamental characteristics. Therefore, the authors aim to apply existing knowledge of anchor–concrete interaction (with concrete considered a brittle material) to the development of the proposed rock detachment technique.

**Figure 3:**
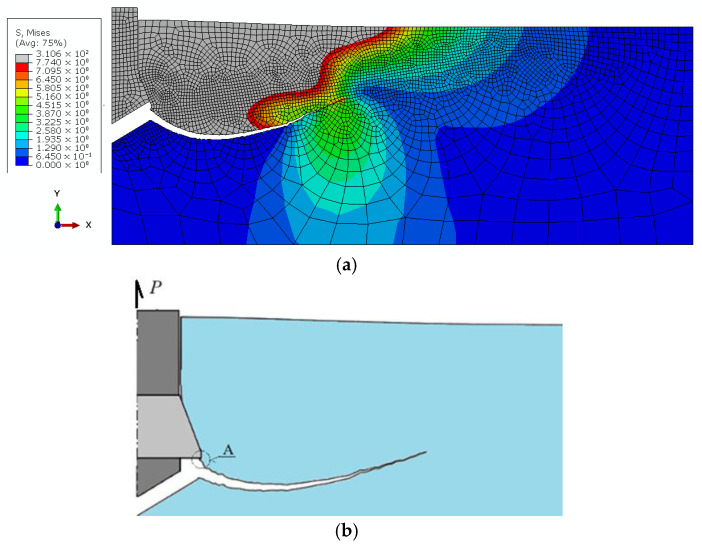
(**a**) Crack propagation trajectory in the medium under the action of a pulled-out undercutting anchor (displacement induced by the pull-out force); (**b**) Crack path during anchor extraction. A—location of the crack initiation point (author’s own work).

As previously mentioned, in typical undercut anchor systems, the failure process is initiated by the action of the anchor head, with the crack initiation zone typically located at the base of the undercutting head, as illustrated in Figure 3. The dominant failure mode involves debonding that originates in this region [73,74].

The location of crack initiation and the trajectory of failure zone propagation are of particular interest to the research team, as they may serve as a key criterion for evaluating the effectiveness of the proposed rock block detachment technology based on anchor pull-out (i.e., maximizing the volume of detached material during anchor extraction).

**Figure 4:**
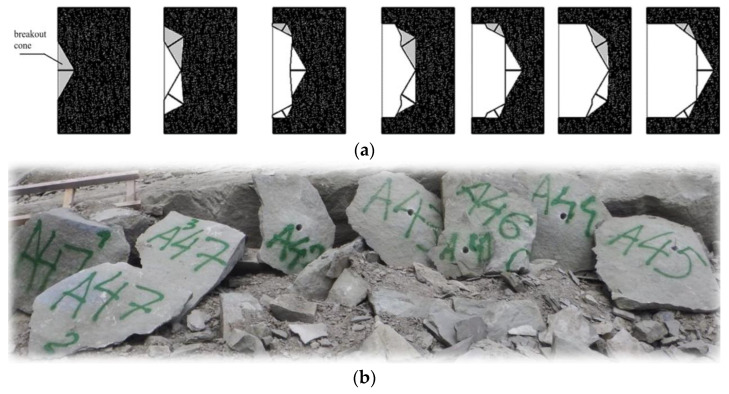
(**a**) Example of applying the proposed technology for local rock detachment at an excavation site; (**b**) Typical rock blocks detached using the tested undercut anchors.

The effectiveness of the proposed detachment technology, in light of the conducted field tests, should be assessed as highly efficient (assuming the implementation of works as specified in the project assumptions). Proportionally to the effective anchoring depth, it is possible to detach blocks of considerable volume (Figure 4 [6,75]).

The design of the undercutting anchor has been repeatedly modified by the authors, along with the associated detachment technology—ranging from the use of standard undercutting anchors for pull-out applications [58,59,76,77] to a redesigned anchor system in which rock detachment is achieved through the expansion of the anchor assembly. In this system, the end of the drive bolt presses against the borehole bottom, while the undercutting head engages a dedicated recess located near the hole base [26,59,73,78,79].

**Figure 5:**
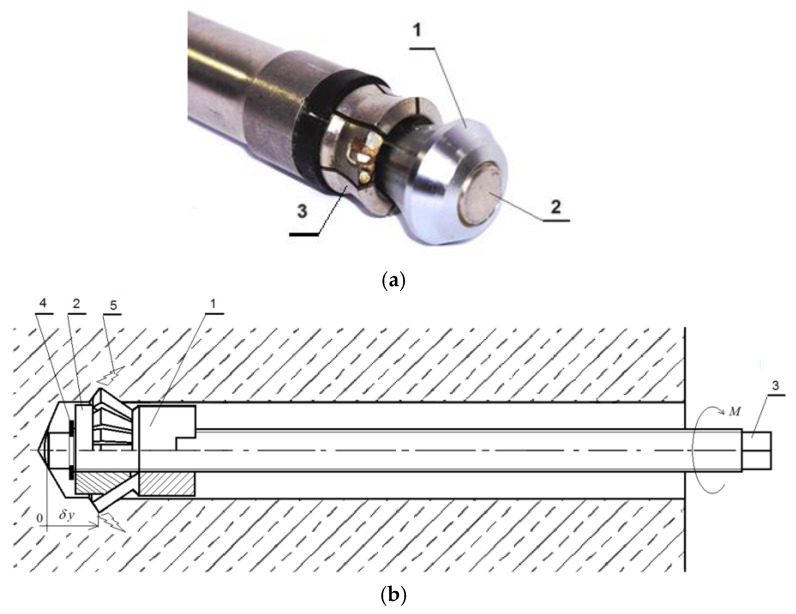
(**a**) Modified HDP-A undercutting anchor used in the initial phase of testing a novel rock detachment technology via tensile-induced fracturing near the borehole bottom: 1—anchor head; 2—drive screw; 3—elastic undercutting sleeve with six cutting segments; (**b**) Modified anchor design for rock mass detachment: 1—undercutting element of the anchor head; 2—conical expansion element; 3—drive screw (anchor core); 4—setting ring; 5—induced fracture in the rock mass; *M*—applied torque on the drive screw; *δy*—imposed linear displacement of the anchor head along the axis of the drive screw.

One of the modified undercutting anchor variants developed specifically for rock block separation, along with the concept of its expansion within the borehole, is illustrated in Figure 5 [26,73].

**Figure 6:**
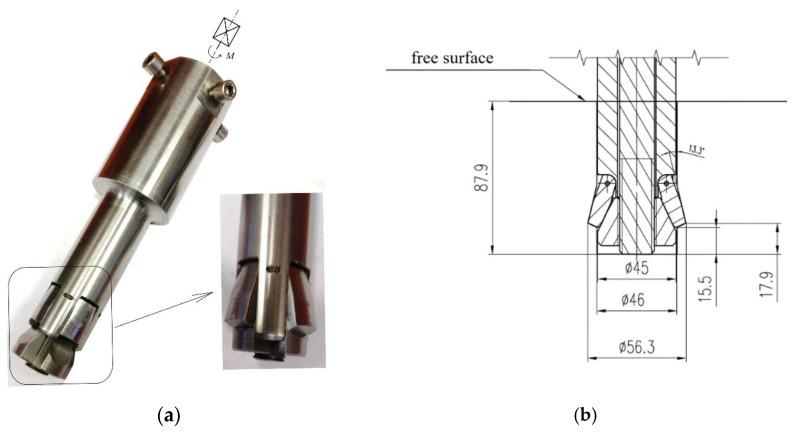
(**a**) Detachment head equipped with a drive torque multiplier; *M*—applied torque; (**b**) characteristic parameters of the anchor.

For the implementation of the novel rock detachment technology, the authors also proposed a dedicated structural solution in the form of a combined undercutting–detachment anchor, designed to enable potential automation of the detachment process (Figure 6, based on [26]).

In the proposed structural solution, both the expansion of the anchor within the undercut borehole and the rock fracturing process leading to detachment are significantly facilitated.

**Figure 7:**
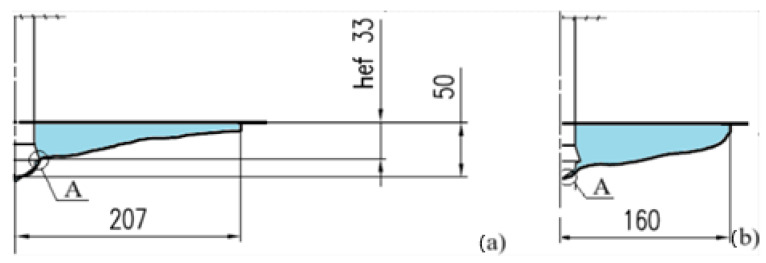
Geometry of the failure zone during the pull-out of an undercutting anchor; A—crack initiation point. (**a**) Initiation located at the base of the conical undercutting head; (**b**) Initiation located at the base of the anchor’s drive screw.

The authors’ research [26,59,78,79] has indicated that, depending on factors such as the geometry of the anchor’s drive screw and the configuration of the borehole bottom in which the anchor is installed, different crack initiation points may occur (see Figure 7).

From the perspective of the proposed rock detachment technology, a critical yet insufficiently explored aspect of using newly designed anchors is the understanding of the failure zone formation mechanism (i.e., the so-called failure cone), including the location of crack initiation and the potential extent of the failure zone (crack propagation trajectory).

The layering, fracturing, and variability in grain size and internal structure of rocks result in anisotropic properties and pose significant challenges for their mathematical description, necessitating the use of advanced computational algorithms to investigate the behavior of such materials under destructive loading [80]. Considering this, as well as the need to determine, for the purposes of the present project, the influence of anchor geometric parameters and process-related parameters on the potential volume of detachment and the extent of spalling (which, to date, has not been analyzed in the literature), the authors decided, at the current stage of research, to adopt the assumption of a homogeneous rock mass structure. This assumption enables a general estimation of the potential damage zone dimensions. In subsequent stages, the influence of rock mass heterogeneity on the investigated failure effects induced by the modified undercut anchor design will be analyzed.

In the present stage of the study, a simplified assumption of a homogeneous and isotropic rock mass was adopted to enable an assessment of the influence of the investigated geometric parameters on crack initiation and propagation mechanisms. The authors acknowledge that, under real geological conditions, sedimentary rocks such as grey sandstone are characterized by the presence of bedding planes, joint sets, and microcracks, which may significantly affect the fracture process [81,82,83,84]. In future research, the effects of rock anisotropy and heterogeneity will be addressed by implementing layered and stochastic models calibrated with field test results.

Despite numerous studies on the fracture mechanics of brittle media under the action of undercutting anchors, most analyses focus on conventional anchor–concrete systems, assuming standard head and drive screw geometries. However, there is a lack of detailed investigations into the influence of the drive screw termination geometry and the shape of the borehole bottom on the initiation and development of the failure zone. Existing numerical models rarely incorporate realistic contact conditions at the interface between the screw and the borehole bottom, nor do they account for the variability in screw termination geometry. This limits their applicability in designing innovative rock detachment technologies.

This research gap is particularly significant in the context of developing non-explosive rock fragmentation methods, where precise control over the location and extent of detachment is crucial for the efficiency and safety of the process.

Therefore, the aim of the present study was to investigate the influence of the drive screw termination geometry in a newly developed undercutting/detachment anchor, as well as the configuration of the borehole bottom, on the location of crack initiation and the potential extent of the failure zone. These factors directly affect the volume of the failure zone and, consequently, the volume of the detached rock mass.

## 2. Materials and Methods

**Figure 8:**
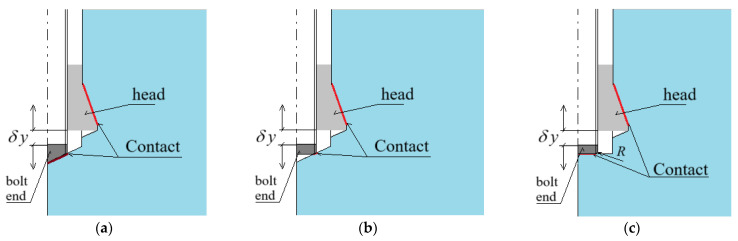
Configurations of the undercutting anchor drive screw termination combined with borehole bottom geometry in rock: (**a**) conical screw end and conical borehole bottom; (**b**) cylindrical screw end with 2 × 2 mm chamfer and conical borehole bottom; (**c**) cylindrical screw end with rounded termination (*R* = 2 mm) and flat borehole bottom (cylindrical hole); *δy*—imposed displacement of the anchor head along the axis of the drive screw.

Typical drive screw termination geometries were considered, including a cylindrical end with a 2 × 2 mm chamfer (a common design in screw manufacturing)—see Figure 8a; and a conical end—see Figure 8b. These configurations are commonly used due to the relative simplicity of their manufacturing processes.

Similarly, the bottom of the borehole is most often conical in conventional applications, as it reflects the profile of the tip of a concrete or soft/medium rock drill bit. However, for the purpose of the present research project, an additional variant was introduced in which the borehole bottom is cylindrical—requiring a specially designed tool with a cylindrical cutting profile.

In one of the tested variants, the standard 2 × 2 mm chamfer was replaced with a rounded termination, featuring a radius of *R* = 2 mm. The combinations of screw termination and borehole bottom shapes considered in the analysis are illustrated in Figure 8.

In practical manufacturing, both the 2 × 2 mm chamfer and the R = 2 mm rounded termination can be produced using standard machining processes, such as CNC turning or milling, with tolerances typically within ±0.1 mm. These geometries are commonly achievable under field workshop conditions using portable machining tools, although ensuring repeatability requires appropriate quality control, e.g., with gauge templates. The selected dimensions were therefore chosen to balance their functional influence on stress distribution with the feasibility of production and installation in mining or geotechnical environments.

The anchor was subjected to a controlled upward displacement along its axis (*Y*-direction). The rock strength parameters used in the numerical simulations fall within the range of values obtained from field investigations and laboratory tests previously conducted by the research team [58,85,86].

The analysis was carried out using the finite element software ABAQUS 2019 [87] (Abaqus 2019, Dassault SystèmesSimulia Corp., Vélizy-Villacoublay, France), with the extended finite element method (XFEM) algorithm applied.

The following damage modeling strategy was implemented in ABAQUS:

**Damage initiation criterion**: Maximum principal stress

**Crack propagation direction**: Perpendicular to the maximum principal tensile stress

**Damage evolution**: Energy-based fracture propagation using a linear softening model

**Material properties of the rock model** were defined as follows:

Young’s modulus: 14,275 MPa

Poisson’s ratio: 0.247

Tensile strength threshold (*f_t_*): 7.74 MPa

Fracture energy (*G_f_*): 0.355 N/m

A stabilization coefficient of 1 × 10^−6^ was introduced to ensure numerical convergence of the XFEM algorithm.

The steel used for the anchor components was modeled as a linear, elastic, and isotropic material, with deformation assumed to remain within the elastic range. The mechanical properties were defined as follows:

**Young’s modulus**: 210,000 MPa

**Poisson’s ratio**: 0.3

**Figure 9:**
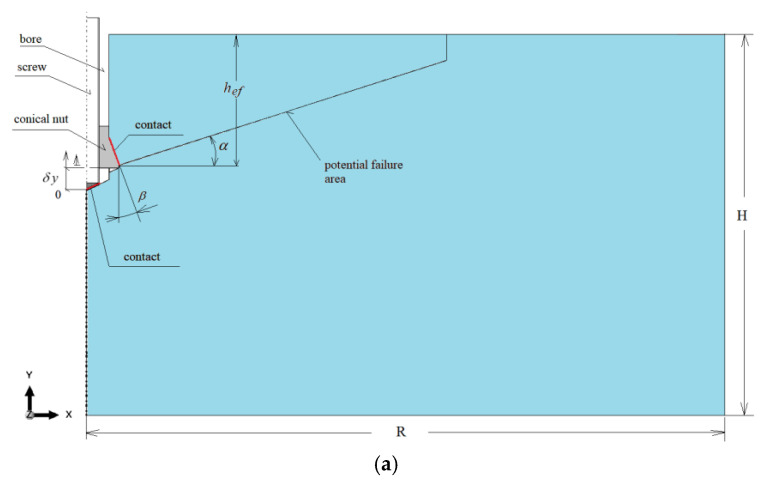

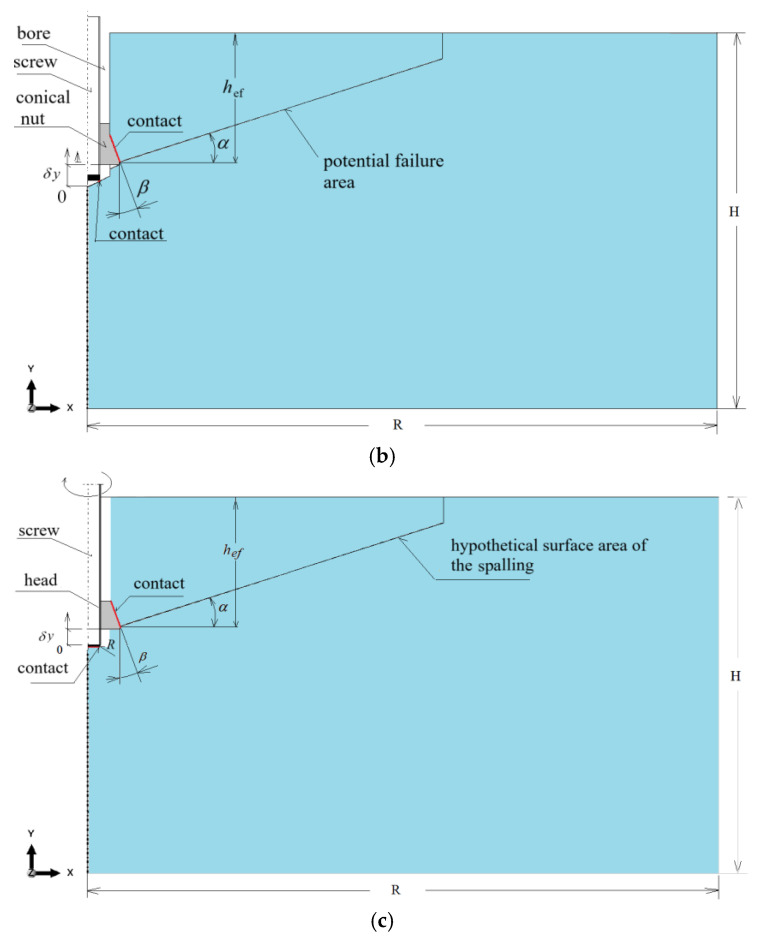
Interaction models of the anchor head with the rock medium: (**a**) conical screw termination with a conical borehole bottom; (**b**) cylindrical screw termination with a 2 × 2 mm chamfer and a conical borehole bottom; (**c**) cylindrical screw termination with a rounded end (*R* = 2 mm) and a flat-bottomed cylindrical borehole.

For the considered anchor termination variants (Figure 8), axisymmetric models of the rock medium with an undercut for the anchor head were developed, as shown in Figure 9. In all cases, the geometry of the undercutting head was identical. Likewise, the effective embedment depth was constant and set to *h*_ef_ = 70 mm.

The dimensions of the model were *R* = 500 mm and *H* = 300 mm. These values were selected to eliminate the influence of boundary constraints on the stress distribution in the rock medium and on the extent of detachment measured on the free surface. The selection of these parameters was based on previous field investigations [58] and numerical analyses [26,59].

The angle α (Figure 9) was defined for auxiliary purposes only, to determine the potential distribution of finite element mesh density used in tracing the fracture trajectory. In existing standards, this angle is typically assumed to be around 35°. However, based on our field studies, it ranges from 13° to 17° for grey sandstones and up to 25° for porphyry [75]. According to our 3D FEM analyses, it can reach values between approximately 27° and 29° [88]. For the purpose of this modeling, an average value of 20° was adopted. In typical anchoring of rock masses, this angle has been observed in the range of 20–27° [54].

**Figure 10:**
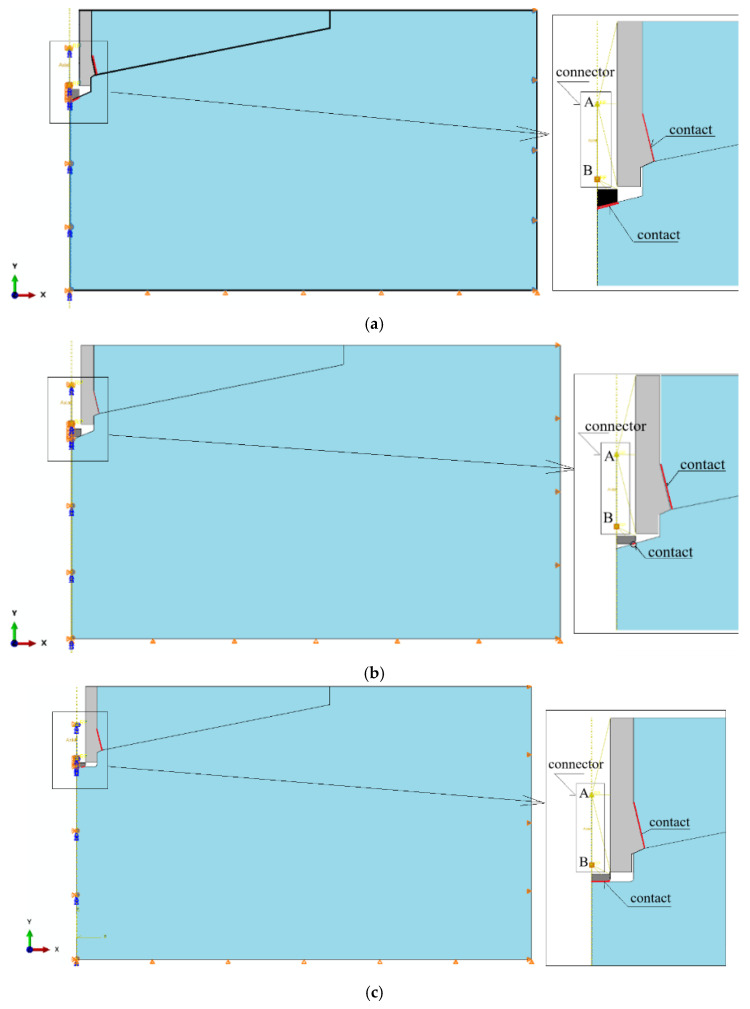
Modeling of the displacement-driven loading of the anchor head using a “Connector” element from the ABAQUS element library; (**a**) conical screw termination with a conical borehole bottom; (**b**) cylindrical screw termination with a 2 × 2 mm chamfer and a conical borehole bottom; (**c**) cylindrical screw termination with a rounded end (R = 2 mm) and a flat-bottomed cylindrical borehole. A, B—node of the connector element.

The interaction between the anchor head and the rock was modeled as an axisymmetric contact problem. Contact was assumed between the conical anchor head and the rock, as well as in the support zone between the termination of the drive screw and the bottom of the borehole (see Figure 9 and Figure 10). In all cases, a constant contact friction coefficient of μ = 0.2 was applied. This value falls within the range of estimates reported in the literature for various steel–concrete and steel–rock interactions [26,59,77,85,89]. For this coefficient value, the project team members obtained good agreement between the crack propagation trajectories recorded during field tests [58] and those from numerical simulations performed using a plane model of the rock medium [90]. Therefore, this value was adopted as the reference point for further numerical simulations. The range of values considered was 0.2–0.6 [26]. Literature data indicate that, for the steel head–rock interface, good agreement between simulation and experimental results is achieved for μ = 0.35–0.4 [91].

As in previous analyses of this problem [78], a displacement-controlled loading condition was assumed. This was realized through the imposed movement of the nut relative to the screw axis and the borehole bottom, achieved by applying a driving torque (*M*, as shown in Figure 5 and Figure 6) to the opposite end of the screw.

The maximum imposed displacement was set to *δy*= 10 mm (as indicated in Figure 9), applied incrementally in discrete steps of 0.01 mm per increment until either mechanical failure occurred or convergence of the computational algorithm was achieved.

To implement this boundary condition, the “Connector Displacement” elements available in the ABAQUS software were used (see Figure 10).

For the analyzed design variants, node A of the connector element was linked to the predefined nodes of the conical anchor head, while node B was connected to the nodes at the end of the drive screw, as shown in Figure 10. By iteratively adjusting the distance between nodes A and B, the vertical separation between the anchor head and the borehole bottom (*δy*, see Figure 8 and Figure 9) was increased along the *OY* axis. This resulted in a progressive deformation of the rock in the anchor contact zone, ultimately leading to fracture initiation and propagation within the rock medium.

The boundary conditions for each rock model were defined as illustrated in Figure 10c. Here, *U* denotes translational degrees of freedom, and *UR* denotes rotational degrees of freedom. The applied constraints were as follows:

**Right edge**: *U_1_* = 0, allowing displacement only in the vertical direction (*Y*-axis)

**Bottom edge**: *U_2_* = 0, allowing displacement only in the horizontal direction (*X*-axis)

**Left edge of the rock domain, left edge of the screw, and connector nodes**: *U_1_* = *U_3_* = *UR_2_* = 0, enforcing axisymmetry with respect to the model’s central axis and allowing displacement only along the *Y*-axis.

**Figure 11:**
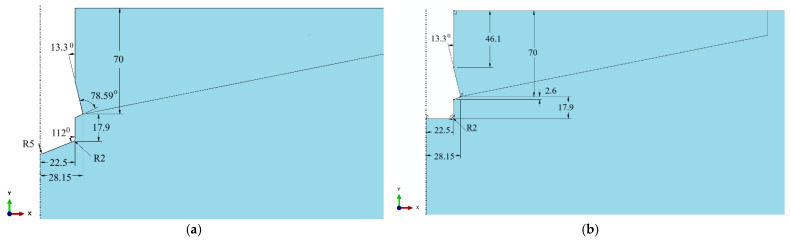
Characteristic dimensions of boreholes corresponding to the investigated variants of the undercutting anchor drive screw: (**a**) conical bottom of the hole, (**b**) flat bottom cylindrical hole.

Figure 11 presents the characteristic dimensions of the undercut profiles in the rock corresponding to the studied borehole bottom configurations.

The rock models with boreholes corresponding to conical and cylindrical screw terminations were assumed to be geometrically identical (Figure 11a), except for the configuration involving a cylindrical screw termination combined with a flat borehole bottom (cylindrical hole), as illustrated in Figure 11b.

**Figure 12:**
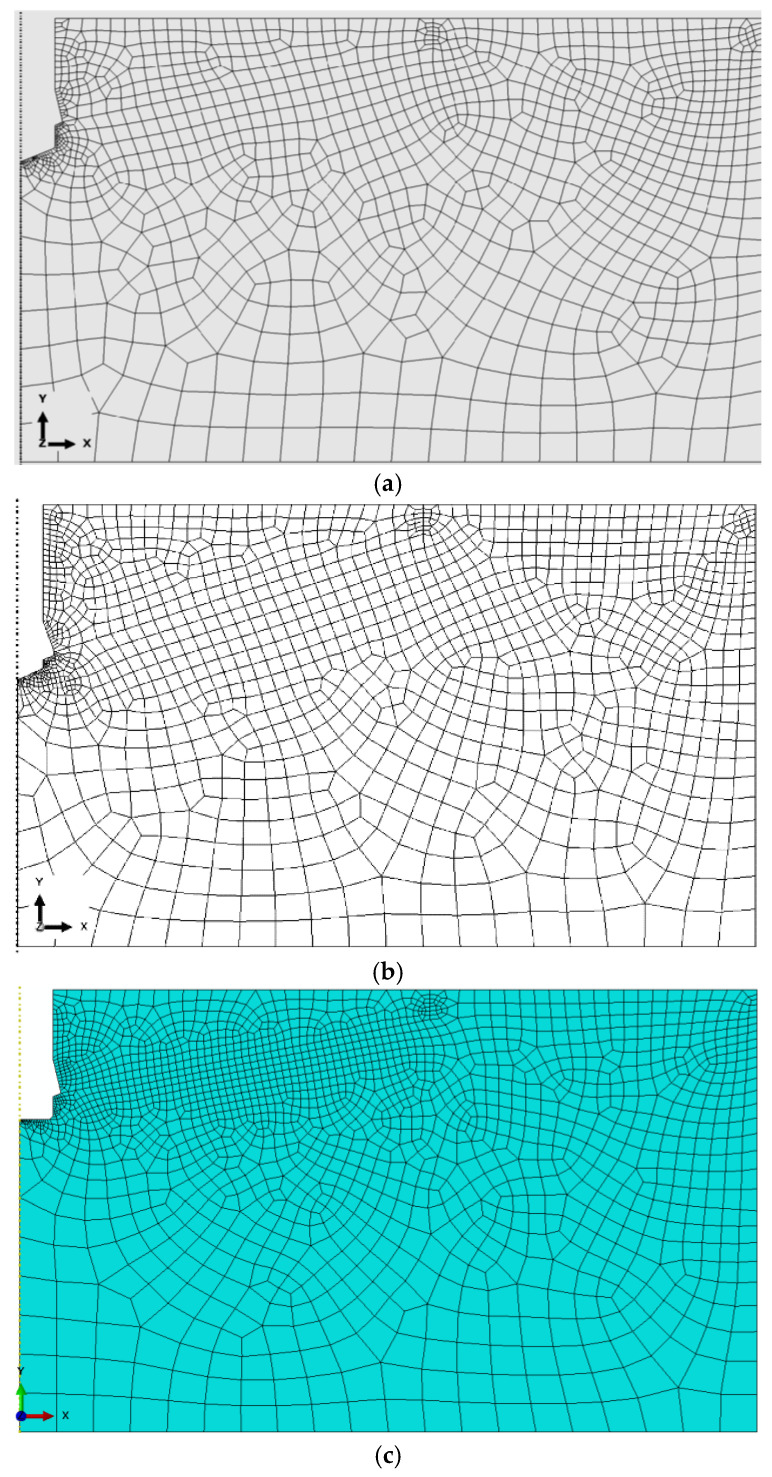
Optimal finite element mesh structures of the rock domain models used in numerical analysis: (**a**,**b**)—conical bottom of the hole, (**c**) flat bottom cylindrical hole.

For each of the developed rock domain models, finite element meshes were generated and are presented in Figure 12. These meshes were obtained based on a mesh sensitivity analysis performed to assess the influence of element size. As this procedure has been repeatedly presented in the authors’ previous publications [73,78,92], only the final mesh configurations are shown here (Figure 12), representing the most efficient solutions in terms of computational time and smoothness of crack surfaces.

The models were discretized using 4-node axisymmetric finite elements of type CAX4R (Continuum, Axisymmetric, 4-node, Reduced integration), generating the finite element mesh shown in Figure 12. This meshing strategy provided an effective compromise between computational efficiency and the accuracy required for predicting stress concentrations and crack initiation in critical regions.

In key regions of the model, a non-uniform mesh density was applied using the automatic mesh generator available in ABAQUS, supplemented by manually defined element seed sizes in selected areas. As a result:

The global element size (edge length) was set to 25 mm;

In the contact zone between the conical anchor head and the rock, the element size was refined to 2 mm;

In the contact region between the screw tip and the borehole bottom, a finer mesh with element sizes ranging from 0.4 mm to 1 mm was used;

Along the anticipated crack propagation path, the mesh was refined to 5 mm to enhance fracture resolution;

Along the upper boundary of the rock domain, the element size varied between 3 mm and 10 mm, depending on the local geometry and loading conditions.

The mesh sensitivity analysis and convergence studies for the XFEM models have been thoroughly presented in the authors’ previous publications [73,78,92]. These studies demonstrated no significant changes in stress distribution or crack propagation trajectories with further mesh refinement, confirming the numerical stability of the adopted approach. Due to the already substantial length of the present paper, the graphical results of these analyses are not repeated here.

The assumptions adopted in the numerical modeling are based on the research team’s prior experience and on literature concerning fracture mechanics of brittle materials and anchor–rock contact behavior. To ensure a proper balance between computational accuracy and efficiency, axisymmetric models were employed using CAX4R-type elements, which enabled precise representation of the contact zone and localization of failure initiation.

The model implemented the extended finite element method (XFEM), allowing for the simulation of crack initiation and propagation without the need to predefine crack paths [93,94]. The damage model was based on the maximum principal stress criterion and energy-based crack evolution, which is suitable for capturing brittle fracture mechanisms characteristic of grey sandstone used in experimental investigations.

Material parameters such as Young’s modulus, tensile strength, and fracture energy were defined based on previous field studies and the authors’ earlier publications, ensuring consistency between the numerical model and experimental observations.

The applied boundary conditions and contact formulation, including a friction coefficient of μ = 0.2 at the interface between the screw and the rock, closely replicate real-world anchor installation conditions. The best correspondence with experimental observations—both in terms of maximum force and fracture pattern—was obtained in the simulation using an anchor with a friction coefficient of 0.2 between the anchor and the rock. However, it was noted that in reality, this coefficient may vary due to changes occurring in the rock during testing [95]. This modeling approach enabled a realistic analysis of the influence of drive screw termination geometry and borehole bottom shape on stress distribution and crack propagation behavior, while substantially reducing the need for costly experimental testing.

## 3. Results and Discussion

This section presents the results of numerical simulations conducted to analyze the influence of the drive screw termination geometry and the borehole bottom shape on the stress distribution and crack propagation trajectory in a rock medium. For each of the investigated model variants, characteristic stress states and failure propagation paths were analyzed under identical loading and material conditions. Particular attention was focused on the contact zone between the anchor screw and the borehole bottom, where the failure mechanism is typically initiated. This analysis is crucial for optimizing the geometric parameters of anchors used in non-explosive rock fragmentation technologies.

To ensure clarity and readability of the results, the data are presented in the form of stress distribution maps and crack propagation visualizations for each geometric configuration. Comparisons were made between drive screw termination types—flat and conical—and borehole bottom shapes—cylindrical and conical. This approach enables a detailed evaluation of the effect of each geometric parameter on the mechanical response of the anchoring system and allows the identification of configurations that promote controlled and efficient fracture propagation.

**Figure 13:**
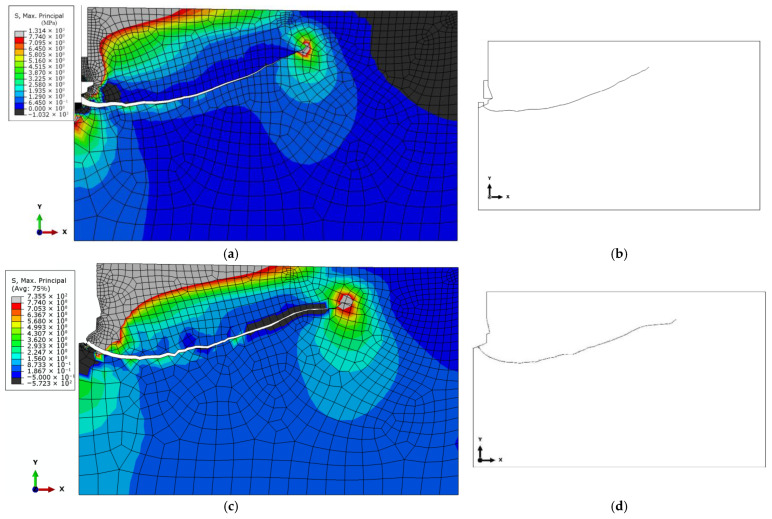

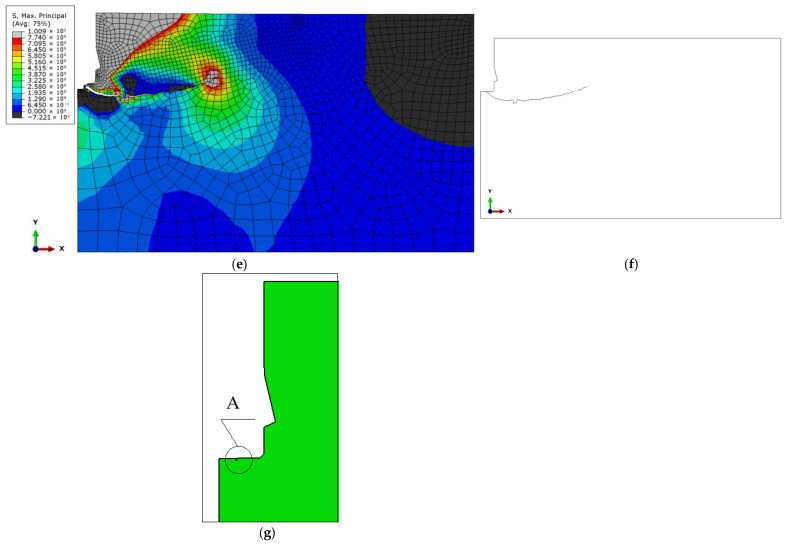
Distribution of maximum tensile stresses (σ_max) and crack propagation trajectories under the action of an undercutting anchor for the analyzed design variants: (**a**) conical screw termination with conical borehole bottom; (**c**) cylindrical screw termination with a 2 × 2 mm chamfer and conical borehole bottom; (**e**) cylindrical screw termination with a rounded end (R = 2 mm) and flat-bottomed cylindrical borehole; (**g**) A—location of the crack initiation point under the action of a cylindrical screw termination (as in the Hertz problem), (**b**,**d**,**f**)—cracking trajectory according to the analyzed model (according to the case of model—(**a**,**c**,**e**)).

As the displacement parameter *δy* (Figure 9) increases, the deformation of the rock under the anchor head also intensifies, eventually leading to the initiation and propagation of a crack at the base of the drive screw, as illustrated in Figure 13.

The results of the analysis demonstrated that, regardless of the drive screw termination geometry, the location of the crack initiation point leading to rock detachment may differ from that observed in conventional pull-out anchor systems (see Figure 3a). For the investigated design variants, fracture was initiated at the base of the drive screw (Figure 13b,d,e). This has direct implications for the volume of the potentially detached rock block, which assumes the form of a so-called pseudo-conical failure zone. The term “pseudo-failure cone” is intentionally used here, as in the case of rocks, the actual shape of the detached fragment deviates significantly from an ideal cone, as discussed in detail in [92].

Analysis of stress distribution and crack propagation trajectories indicates a significant influence of both the drive screw termination geometry and the borehole bottom shape on the initiation and development of the failure zone in the rock medium. In the case of a flat screw termination with a 2 × 2 mm chamfer combined with a conical borehole bottom, strong stress concentrations were observed in the contact zone, leading to the formation of a single dominant crack path. This trajectory penetrates deepest into the material, favoring the detachment of a larger rock volume.

In contrast, the remaining configurations resulted in shallower crack paths that reached the free surface of the rock more quickly. The simulation results suggest that it is potentially feasible to control the stress distribution by appropriately selecting the shape of the drive screw termination in the detachment anchor. Consequently, it becomes possible to control, to some extent, the extent and volume of the detached rock mass.

The analysis results also confirmed the premises of preliminary investigations [26,79], in which a deviation from typical crack propagation modes was observed during the failure of rock structure using one of the newly developed detachment anchor variants. Specifically, an alternative initiation and propagation pattern of the fracture was noted, differing from that observed in conventional anchoring systems.

These findings are of significant importance for the advancement of rock detachment technologies utilizing undercutting anchors. In particular, the use of drive screws with specially designed terminations may enable the generation of failure with a controlled character by enforcing specific crack propagation paths within the rock medium. This is especially relevant in underground excavation environments, where control over fracture direction directly affects both the safety and efficiency of the process.

The results suggest that a properly designed anchor termination geometry can reduce the need for explosives, thus contributing to the development of more environmentally friendly and selective rock fragmentation methods.

The qualitative analysis performed in this study confirmed the presence of all three primary fracture modes (I–III) during the rock detachment process. In future research stages, the numerical model will be extended to include a quantitative decomposition of the fracture energy into individual modes, enabling a mode mixity analysis and a more precise assessment of the influence of the screw termination geometry on the dominant failure mechanism. This approach will facilitate better tailoring of the anchor design to achieve the desired crack propagation direction and character.

Although the present study primarily focused on the influence of drive screw termination geometry and borehole bottom shape on crack initiation and propagation, the numerical results indirectly provide insight into the characteristics of the damage zone preceding visible fracture formation. The observed stress concentration patterns and early-stage crack initiation points correspond to the onset of a localized fracture process zone (FPZ), the extent of which can be qualitatively inferred from the XFEM stress–strain fields. Future work will incorporate a continuum damage mechanics (CDM) approach to quantitatively capture FPZ evolution and its role in determining the size and morphology of the detached rock fragment.

It should be emphasized; however, that the presented analyses are based on a numerical model that—despite incorporating realistic boundary conditions and material properties—does not account for all factors present in real operating environments; such as natural fractures; structural heterogeneity of the rock; or localized material damage. Future research should include model calibration using extended experimental datasets, including field trials and destructive testing, to validate the performance of different screw termination geometries under actual working conditions.

It is also recommended to consider the application of nonlinear fracture mechanics methods and heterogeneous material models for further optimization of undercutting anchor designs.

In light of the obtained results, the growing role of advanced computational methods—such as the Finite Element Method (FEM) [96,97,98,99]; the Boundary Element Method (BEM) [100,101,102,103,104,105]; and artificial neural network (ANN)-based models [106,107,108,109,110,111]—should be highlighted in engineering analysis of complex contact problems. The use of such tools enables a deeper understanding of the mechanisms governing crack initiation and propagation in brittle materials while also significantly reducing the need for costly and time-consuming experimental testing in the early design phase.

Properly calibrated numerical models allow for extensive parametric studies that support the optimization of anchor geometry and their adaptation to variable operating conditions. Moreover, the integration of machine learning algorithms may, in the future, contribute to the automation of identifying key geometric and material features that influence rock detachment effectiveness—representing an important step toward the development of intelligent anchoring system design.

In this study, the numerical analyses were conducted under the assumption of a homogeneous, isotropic rock medium without pre-existing fractures. These simplifications allowed for a clearer evaluation of the influence of anchor geometry and load distribution but represent an idealized scenario. In real rock masses, heterogeneity of lithology, variable joint patterns, and the presence of natural discontinuities can significantly affect load transfer mechanisms and failure propagation. Therefore, the direct applicability of the presented results to insitu conditions should be approached with caution. Future research will include models incorporating spatial variability of material properties and pre-existing fracture networks to better reflect the complexity of natural rock masses.

### 3.1. Experimental Validation

**Figure 14:**
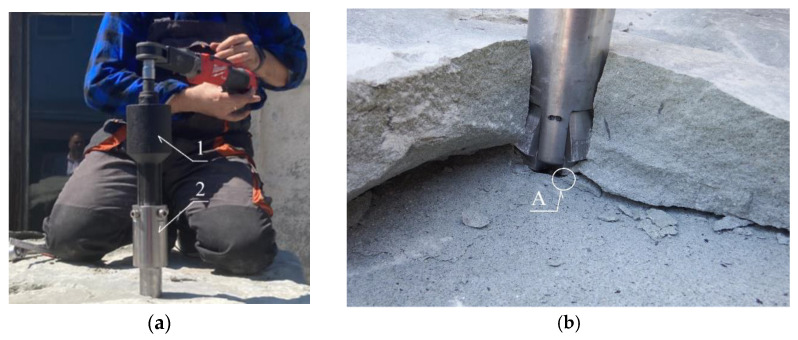
(**a**) Rock detachment using the newly developed undercutting anchor (2), assisted by a torque multiplier (1); (**b**) Detached sandstone block in the form of a pseudo-failure cone; A—location of the crack initiation point at the base of the drive screw.

Experimental studies on rock block detachment using undercutting anchors—both with the modified HDP-A anchor variant (as shown in Figure 3a) and with the newly developed anchor design [78]—were conducted either in the Brenna sandstone quarry (OPUS 10 competition No. 2015/19/B/ST10/02817, financed by the Polish National Science Centre)or at the test facility located at ITG KOMAG. In the latter case, tests were performed on sandstone blocks originating from the Brenna quarry, as illustrated in Figure 14.

To enhance the reliability of the numerical model, preliminary experimental tests were repeated three times for each configuration, ensuring result repeatability within ±7% of peak load values. The measurement system uncertainty was determined for the applied force and displacement sensors. Although full stress–strain curves were not recorded for all trials, representative results illustrating the load–displacement relationship and the corresponding crack initiation and propagation sequences were obtained. Additionally, high-resolution photographs captured during testing document the onset and development of fractures, enabling direct visual verification of numerical predictions.

The tested rock was grey sandstone (a sedimentary rock), with mechanical properties summarized in Table 1.

The sandstone exhibited a layered structure, with individual strata approximately 0.7–1.0 m thick, as described in detail in [112]. The tests were conducted in a homogeneous section of the rock mass, free from fractures or structural disturbances.

For the newly developed anchor design (item 2 in Figure 14a), the experiments were performed using a torque multiplier (item 1 in Figure 14a).

The field tests generally confirmed that the initiation point of the detachment-inducing crack is located at the base (termination) of the drive screw (point A, Figure 14b). This location corresponds to the contact zone between the drive screw and the bottom of the borehole in which the anchor is installed.

### 3.2. Model Limitations and Future Research Perspectives

**Figure 15:**
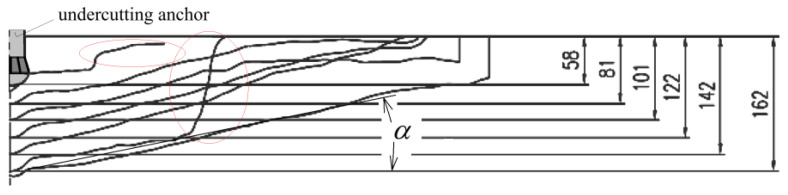
Disturbances in crack propagation trajectories in a hypothetically homogeneous grey sandstone.

It should be noted that the premises of the present analysis involve several limitations, arising from the following factors:

- The use of a homogeneous rock medium model, selected to best represent the mechanical properties of grey sandstone from the Brenna quarry. This represents an idealized case, as natural rocks often exhibit fracturing, moisture presence, or internal heterogeneity [37,54,58];
- The application of a linear elastic material model, which is a significant simplification considering the wide variability in internal structure and deformation behavior across different rock types [80]. Disturbances in crack propagation trajectories (Figure 15) were observed even in hypothetically homogeneous grey sandstone from the “Zalas” mine (based on [59]).

- The use of an undercutting anchor with a conical head, which introduces a load transfer mechanism that differs significantly from that of bonded or cast-in-place anchors [12,55,78,92];
- The assumed installation method, including anchor expansion and interaction with the rock medium, differs from the conventional behavior of pull-out-type undercutting anchors.

**Figure 16:**
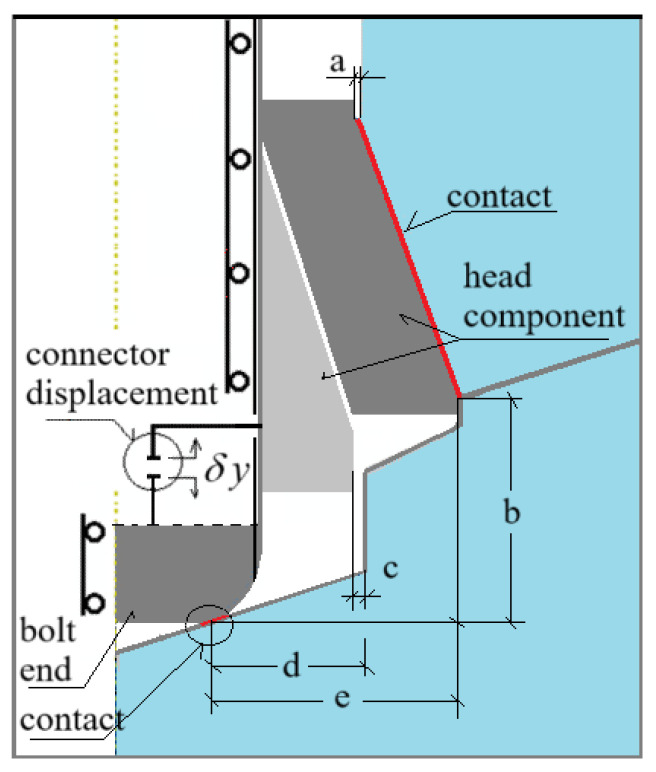
Geometric relationships relevant to the simulation outcome in the model of detachment anchor interaction with the rock medium.

Moreover, there are indications that the outcomes of the numerical simulation may also be significantly influenced by the following factors:

- Substantial variability in the mechanical properties of rock media, including Young’s modulus, Poisson’s ratio, Coulomb friction coefficient at the anchor–rock interface, moisture content, stratification, and natural fracturing of the rock mass;
- The combination of geometric parameters of the mechanical model of the rock medium (Figure 16) and the anchor head geometry—particularly the clearance dimensions (*a* and *c*)—as these may alter the direction and distribution of force transmission from the anchor to the rock [113];
- The detachment process in grey sandstone, being a sedimentary rock, may depend on the orientation of the bedding planes. In the present study, the borehole axis was oriented predominantly perpendicular to the sedimentation/bedding planes.

**Figure 17:**
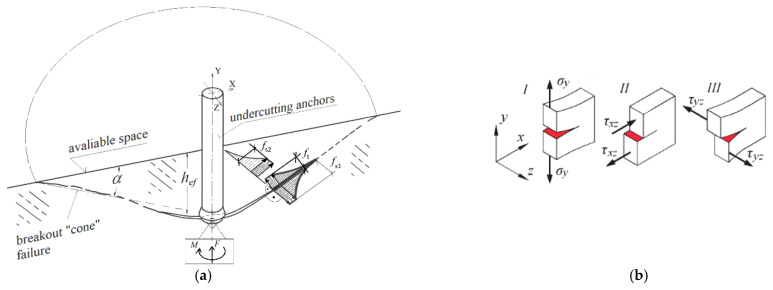
(**a**) Complex fracture model of the rock medium under the action of a conical anchor head, accounting for torsional friction between the rotating head and the rock; (**b**) Fundamental fracture modes in continuous media. *M*—friction torque; *F*—applied load/force.

It should also be noted that in the proposed anchor design, the rotational motion of the drive screw within the conical head introduces tangential (circumferential) friction forces acting on the conical surface (in a full 3D interaction model). This significantly alters the mechanical conditions compared to simplified assumptions commonly used in planar models (e.g., cohesive zone models) or axisymmetric models. A third fracture mode emerges (see Figure 17), more complex than those considered in previous studies [26,79], particularly in models developed for pull-out failure of installation-type anchors (flat 2D models [90,114]).

Although the present study applied a maximum principal stress criterion with linear softening—suitable for modeling brittle fracture initiation in homogeneous sandstone—the authors acknowledge that complex mixed-mode (I/II/III) fracture mechanisms may occur under the combined tensile; shear; and torsional loading conditions generated by the novel anchor design. Future work will therefore incorporate more advanced fracture modeling approaches, such as cohesive zone models (CZM) with traction–separation laws, J-integral-based fracture parameter evaluation, and crack tip stress intensity factor (SIF) analysis, to more accurately capture multi-mode crack propagation in heterogeneous rock masses. These developments will allow for improved calibration of numerical results against experimental field data.

The contact between two surfaces in relative rotational motion is a well-known phenomenon in many engineering applications [115,116], and the resulting torsional friction contact is a critical aspect in this case.

The action of the pull-out force *F* (Figure 17a) induces Mode I fracture (opening/tensile failure). The conical shape of the anchor head introduces deformations in the X-direction of the model and leads to Mode II fracture (in-plane shear along the XZ plane). Additionally, the torque *M* applied to the drive screw causes rotational movement of the head under friction, potentially generating Mode III fracture (out-of-plane shear in the YZ plane).

Most existing models are based on classical pull-out tests of foundation-type anchors with cylindrical heads, for which cohesive zone modeling is justified. However, in the case of the pull-out behavior of the anchor configuration considered in this study, such simplification is insufficient and may not capture the full complexity of the failure mechanism.

Although the axisymmetric modelling approach adopted in this study enabled a detailed analysis of the interaction between the anchor head and the rock medium, it inherently cannot capture asymmetric fracture patterns or complex 3D crack propagation, particularly under the influence of torsional forces that may induce Mode III shear. In real operating conditions, such effects can result in non-axisymmetric crack trajectories, especially in heterogeneous rock masses with natural discontinuities. Therefore, future work will involve the development of fully 3D numerical models to account for these additional fracture modes and to provide a more comprehensive representation of the rock detachment process.

## 4. Conclusions

Given the number of potentially influential factors, it is essential to conduct more extensive field investigations encompassing a broader diversity of rock structures, as well as an in-depth parametric analysis. This should include key geometric parameters of both the anchor and the undercut borehole—extending beyond the scope of previous studies [26,75,78,79].

Although the present study focuses on macroscopic FEM-based simulations using XFEM to capture crack initiation and propagation, it does not explicitly model the micro-to-macro transition of fracture development. Future work will address this gap by integrating approaches from damage mechanics and discrete fracture network (DFN) modelling, which enable the explicit representation of micro-crack nucleation, coalescence, and their evolution into macro-scale fractures. Such integration is expected to provide deeper mechanistic insight into the fracture process and improve the predictive capability of the numerical models.

## 5. Summary and Conclusions

Field investigations confirmed that, for the tested rock type (medium-strength grey sandstone), the location of the crack initiation point during detachment with anchors expanded near the borehole bottom is consistently found in the contact zone between the screw tip and the borehole bottom—regardless of the screw termination variant. This differs from typical undercutting anchors pulled out from the substrate, where fracture initiation commonly occurs at the base of the conical head (as shown in Figure 3).

The conducted analyses demonstrate that the geometry of the screw termination and the shape of the borehole bottom play a critical role in the initiation and progression of failure in the rock medium. Cylindrical terminations with a chamfer (as shown in Figure 8) generate deeper crack penetration compared to conical or cylindrical terminations combined with flat-bottomed boreholes. This may be advantageous in the context of detaching larger volumes of rock material under lower axial loading forces.

These findings provide a foundation for the design of new types of undercutting anchors dedicated to rock detachment applications. Such designs could be optimized for directional crack propagation and efficient fragmentation without the use of explosives.

The developed anchor configurations may find direct application in a variety of engineering scenarios where controlled, non-explosive rock detachment is required. Potential use cases include tunneling in urban or environmentally sensitive areas, selective rock removal in mining operations to minimize dilution, and stabilization of rock slopes or excavation fronts in geotechnical engineering. In such contexts, the ability to control crack initiation and propagation through optimized anchor geometry could significantly improve both operational safety and process efficiency.

The presented numerical models—once properly calibrated with experimental data—may serve as valuable tools for supporting the development of selective rock detachment technologies in underground mining and geotechnical engineering.

## Figures and Tables

**Table 1:** Mechanical properties of grey Brenna sandstone.

Mine	*f*_c_ (MPa)	Standard Deviation *f*_c_	*f*_t_ (MPa)	Standard Deviation *f*_t_	*k* = *f*_c_/*f*_t_	*φ* (°)	c (MPa)	Rock	Description
Brenna	58.8	9.29	3.9	1.17	15.1	53	6.0	sandstone	Sandstone layered, weak

## Data Availability

The original contributions presented in this study are included in the article. Further inquiries can be directed to the corresponding author.
